# Natural killer cell biology and therapy in multiple myeloma: challenges and opportunities

**DOI:** 10.1186/s40164-024-00578-4

**Published:** 2024-11-13

**Authors:** Kamlesh Bisht, Aimee Merino, Rob Igarashi, Laurent Gauthier, Marielle Chiron, Alexandre Desjonqueres, Eric Smith, Edward Briercheck, Rizwan Romee, Evren Alici, Eric Vivier, Michael O’Dwyer, Helgi van de Velde

**Affiliations:** 1 Research and Development, Sanofi, Cambridge, MA 02141 USA; 2 Division of Hematology, Oncology, and Transplantation, University of Minnesota, Minneapolis-Saint Paul, MN USA; 3 Innate Pharma Research Laboratories, Innate Pharma, Marseille, France; 4 Research and Development, Sanofi, Vitry-sur-Seine, France; 5 Division of Hematologic Malignancies and Transplantation, Dana Farber Cancer Institute, Boston, MA USA; 6 Department of Medicine, Karolinska Institutet (KI), Huddinge, Sweden; 7 Centre d’Immunologie de Marseille-Luminy, Aix Marseille Université, CNRS, INSERM, Marseille, France; 8 Marseille-Immunopôle, APHM, Hôpital de la Timone, Marseille, France; 9 Department of Haematology, University of Galway, Galway, Ireland

**Keywords:** Multiple myeloma, Natural killer cells, Natural killer cell activating receptor, Natural killer cell inhibitory receptor, Immunomodulation, Natural killer cell engagers

## Abstract

Despite therapeutic advancements, multiple myeloma (MM) remains incurable. NK cells have emerged as a promising option for the treatment of MM. NK cells are heterogenous and typically classified based on the relative expression of their surface markers (e.g., CD56 and CD16a). These cells elicit an antitumor response in the presence of low mutational burden and without neoantigen presentation via germline-encoded activating and inhibitory receptors that identify the markers of transformation present on the MM cells. Higher NK cell activity is associated with improved survival and prognosis, whereas lower activity is associated with advanced clinical stage and disease progression in MM. Moreover, not all NK cell phenotypes contribute equally toward the anti-MM effect; higher proportions of certain NK cell phenotypes result in better outcomes. In MM, the proportion, phenotype, and function of NK cells are drastically varied between different disease stages; this is further influenced by the bone marrow microenvironment, proportion of activating and inhibitory receptors on NK cells, expression of homing receptors, and bone marrow hypoxia. Antimyeloma therapies, such as autologous stem cell transplant, immunomodulation, proteasome inhibition, and checkpoint inhibition, further modulate the NK cell landscape in the patients. Thus, NK cells can naturally work in tandem with anti-MM therapies and be strategically modulated for improved anti-MM effect. This review article describes immunotypic and phenotypic differences in NK cells along with the functional changes in homeostatic and malignant states and provides expert insights on strategies to harness the potential of NK cells for improving outcomes in MM.

## Background

Multiple myeloma (MM) is the second most common hematologic malignancy in the United States, with over 32,000 new cases in 2020. It is characterized by monoclonal expansion of terminally differentiated plasma cells [1]. Before developing active myeloma, patients undergo two asymptomatic precursor disease stages, namely monoclonal gammopathy of undetermined significance (MGUS) and smoldering multiple myeloma (SMM), which are characterized by the presence of an M-protein in the serum and/or excess bone marrow (BM) plasma cells [2]. Despite numerous therapeutic advancements and concomitant increase in survival outcomes, MM remains an incurable disease [1].

Recent studies have focused on NK cells as a promising therapeutic avenue for improved MM outcomes. NK cells are involved in the defense against viral-infected and malignant cells [3–7]. They are effector innate lymphoid cells (ILCs), which constitute 2–15% of total lymphocytes in humans. NK cells recognize and eliminate major histocompatibility complex-I (MHC class I) low or negative malignant and virally infected host cells [8, 9]. Human NK cells are usually classified into two categories based on the expression of the two surface markers cluster of differentiation (CD)56 (encoded by neural cell adhesion molecule [*NCAM*)] and CD16a (encoded by Fc gamma receptor IIIa [*FCGR3A*] [10]. CD56^bright^CD16^−^ NK cells produce cytokines, growth factors, and chemokines but show lower cytotoxicity than CD56^dim^CD16^+^ NK cells, which produce granzymes and perforin (PRF1) as well as growth factors, cytokines, and chemokines [11, 12]. NK cell number and function correlate with survival outcomes; however, their proportion and phenotype vary by MM stage and may be negatively impacted by antimyeloma treatments. Conversely, many drugs against MM rely on the activation or recruitment of NK cells [11, 13, 14].

Both natural killer (NK) and CD8^+^ T cells are cytotoxic effector cells with intrinsic antiviral and antitumor capabilities; however, the recognition mechanisms of NK cells are different from those of T cells [11, 15]. T cell activation is led by T cell receptors [15, 16], whereas NK cell activation is dependent on an equilibrium between an array of germline-encoded innate activating and inhibitory receptors [11]. Moreover, T cells can be activated by even a single peptide-MHC 1 (p-MHC I) [15, 16], whereas NK cells recognize specific MHC I molecules through inhibitory receptors; moreover, NK cells recognize self-molecules that are upregulated on stressed cells; this process is called stress-induced self-recognition, which results in the elimination of non-self or transformed cells [11]. A recent classification classified NK-cells into three subtypes NK-1, NK-2 and NK-3 based on single-cell RNA sequencing (scRNA-seq) and cellular CITE-seq data. The molecular characteristics, key transcription factors expression and biological functions and cytokine responses of these subgroups are distinct, and this study adds to our understanding of NK cell diversity [17].

Thus, NK cell-based therapies hold immense potential in improving MM outcomes; understanding NK cell biology, NK cell–MM interaction, and effect of existing antimyeloma agents on NK cells is crucial for designing new NK cell-based therapies. This review article provides an overview of NK cell biology in MM, outlines the current therapeutic landscape of MM and the associated challenges, and provides expert opinion on harnessing NK cells for MM treatment.

## NK cells in MM

### NK cell phenotype and status in MM

**Fig. 1:**
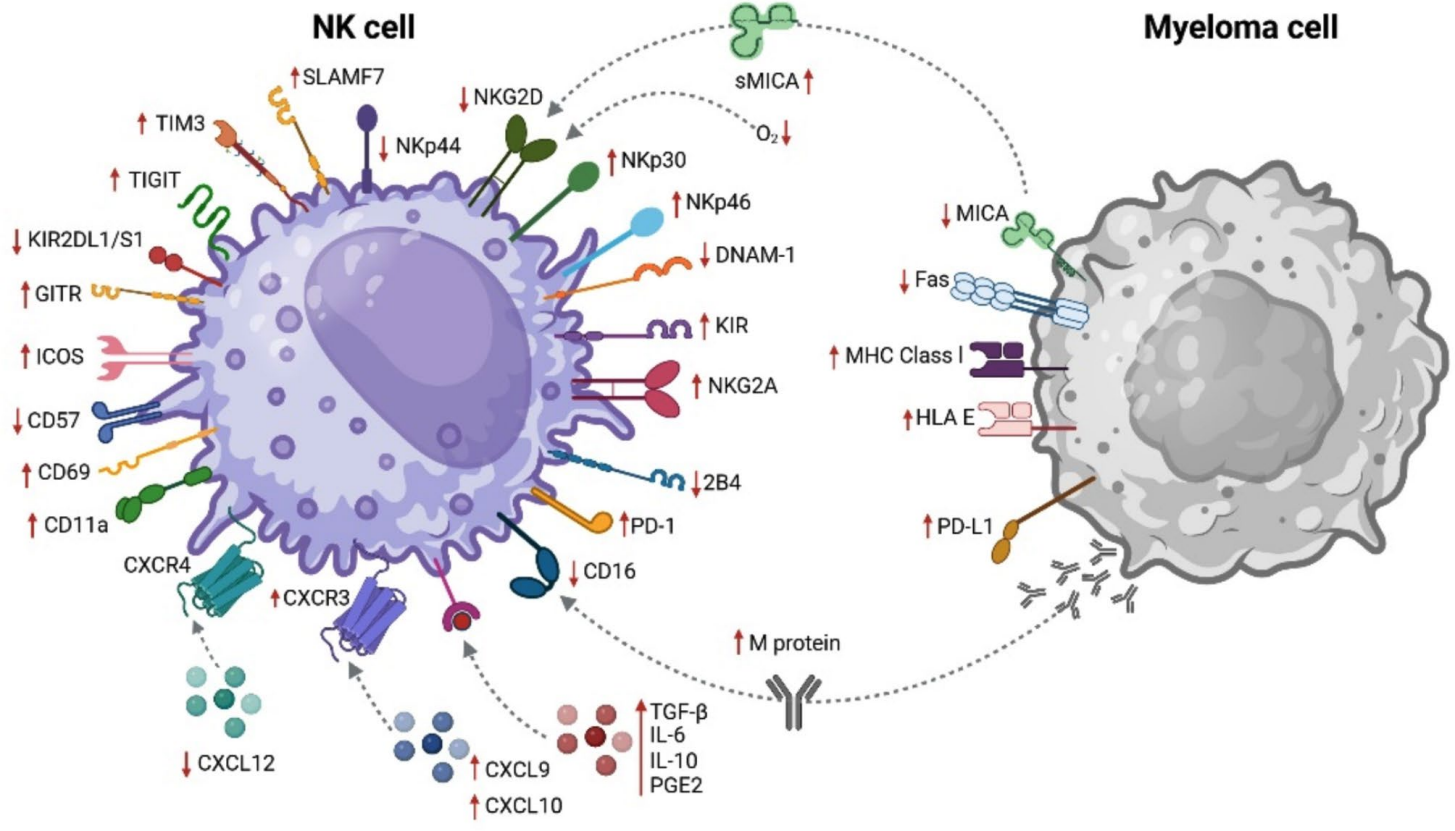
Effect of MM on NK cells: Significantly increased (↑) or decreased (↓) expression of surface markers on NK cells and RRMM cells [13]. **CCL**, CC-chemokine ligand; **CCR**, chemokine receptor; **CD**, cluster of differentiation; **CXCL**, CXC motif ligand; **CXCR**, CXC chemokine receptor; **CX**_**3**_**CR**, CX3C motif chemokine receptor; **DL**, domain long cytoplasmic tail; **DNAM-1**, DNAX accessory molecule-1; **DS**, domain short cytoplasmic tail; **GITR**, glucocorticoid-induced TNFR-related protein; **HLA**, human leukocyte antigen; **ICOS**, inducible T cell costimulatory; **IL**, interleukin; **KIR**, killer immunoglobulin like receptors; **MHC**, major histocompatibility complex; **MM**, multiple myeloma; **NK**, natural killer cell; **PD-1**, programmed cell death protein 1; **PD-L1**, programmed death ligand 1; **PGE2**, prostaglandin E2; **RRMM**, relapsed/refractory multiple myeloma; **sMICA**, soluble MHC class I chain-related gene A; **SLAMF 7**, signaling lymphocyte activation molecular family 7; **TGF-β**, transforming growth factor beta; **TIGIT**, T-cell Ig and ITIM domain; **TIM3**, T cell immunoglobulin

The immunophenotypic landscape, including NK cell subsets and surface markers—inhibitory and activating receptors, are markedly altered in MM (summarized in Fig. 1).

#### NK cell subsets in MM

Development and maturation of NK cells is a continuous process that begins in the BM and proceeds to peripheral tissues, where NK cells acquire various activating and inhibitory receptors and mature into functional cells. Understanding the functional differences between and prognostic implications of various NK cell subsets in MM is crucial for developing targeted immunotherapies and monitoring disease progression. Certain NK cell subsets, such as CD56^+^CD3^−^ and CD57^+^CD8^−^ subsets, are associated with adverse prognosis and better outcomes, respectively, in patients with MM. MM growth can affect the functions and migratory properties of effector NK cells, potentially impairing their antitumor response [18]. Several prominent changes in the BM NK cells have been reported in relapsed/refractory multiple myeloma (RRMM). Moreover, in the BM of patients with MM, differential expression of surface markers of various NK cell subsets was reported, i.e., reduced expression of activating receptors, DNAX accessory molecule-1 (DNAM-1), NKG2D, CD16, and NKp44, which could contribute to attenuated antitumor response [13]. The percentage of CD56^+^ cells was also found to be significantly increased in the BM of patients with newly diagnosed multiple myeloma (NDMM), whereas the proportion of CD16^+^ cells did not differ significantly from that of normal BM samples [19]. BM NK cells from patients with MM also expressed high levels of T cell immunoglobulin and mucin domain-3 (TIM-3) [11]. NK cells with TIM-3 blockade exhibited increased cytolytic activity against primary MM cells, which was associated with upregulation of cytotoxicity-related molecules, such as PRF1, granzyme B, tumor necrosis factor-α (TNF-α), and interferon-γ (IFN-γ) [20].

The specific antimyeloma effect of distinct NK cell subsets remains unclear. During MM progression, cytolytic activity of CD56^dim^CD16^−^ NK cells is higher than that of CD56^bright^CD16^+/–^ and CD56^dim^CD16^bright^ NK cells against MM cells with impaired activity and decreased DNAM-1 expression. CD56^dim^CD16^−^ NK cells exhibit faster recovery after hematopoietic stem cell transplantation (HSCT) [21]. However, NK cells are exhausted upon chronic encounters with MM cells, especially the CD56^dim^ subset, followed by impaired proliferation, programmed cell death protein 1 (PD-1) upregulation, and loss of TIM-3 expression [13, 22]. Although the proportion of NK cells is unaltered during progression from MGUS to SMM and MM, immunophenotypic differences in NK cells are observed; significantly lower proportions of CD57^–^CD56^+^ and CD57^–^CD16^+^ lymphocyte subsets are observed in patients with MM than in those with MGUS [23]. Increased CD16 and CD57 levels and CD56^+^ lymphocyte numbers are reported in peripheral blood (PB) and BM samples of untreated patients with MM, respectively, compared with those of matched healthy volunteers. Higher proportion of CD56^+^CD3– cells was significantly associated with poor prognosis and several adverse prognostic factors (anemia, hypoalbuminemia, renal failure, high β2 microglobulin level, DNA diploidy, and high S-phase plasma cell count), whereas higher CD57^+^CD8– cell number correlated with better outcome; this correlation was not observed for CD16^+^ cells [19]. This could be because MM cells colonize the BM and affect the normal ontology and maturation of NK cells [19].

#### Activating receptor expression

Natural cytotoxicity triggering receptor 2 (NCRs) and NKG2D receptors on NK cells play an important role in myeloma disease progression. NKG2D downregulation is associated with progression of MGUS to MM. MM cells can significantly reduce NKG2D receptor expression on NK cells, which is mainly mediated via MM–NK cell interaction and high MHC class I polypeptide-related sequence A (MICA) expression [24]. The immune escape mechanism of MM cells involves NKG2D induction and CD244/2B4/p38 downregulation on NK cells [24, 25]. In patients with RRMM and post-stem cell transplantation (pSCT), NK cells are less mature and express lower levels of DNAM-1, NKG2D, and CD16; and higher levels of TIM3, T-cell Ig and ITIM domain (TIGIT), inducible T cell costimulatory (ICOS), and glucocorticoid-induced TNFR-related protein (GITR) than those of healthy controls; NKp46 expression is maintained or slightly increased in both CD56^dim^ and CD56^bright^ NK cells. This differential expression is more pronounced in BM NK cells than in PB NK cells, suggesting NK cell exhaustion upon chronic encounters with MM cells. In patients with NDMM, high expression of signaling lymphocytic activation molecule family 7 (SLAMF7) on mature and immature NK cells correlates with poor progression-free survival (PFS). BM NK cells are less mature and express lower levels of activating receptors DNAM-1, NKG2D, and CD16 in patients with RRMM and pSCT than in healthy controls [13].

Overall, DNAM-1, NKG2D, and CD16 are key NK cell receptors, which are downmodulated as the disease progresses and are associated with unfavorable outcomes in MM.

#### Inhibitory receptor expression

Malignant plasma cells express human leukocyte antigen-I (HLA-I), which confers protection by engaging inhibitory killer immunoglobulin-like receptors (KIRs) and CD94/NKG2A and by preventing NK cell-mediated lysis [22, 26]. Increase in the levels of CD158a receptor, an inhibitory KIR, and decrease in CD161 receptor levels are responsible for impaired antitumor activity of NK cells in patients with MM [27]. Minimal residual disease (MRD)^+^ patients have lower NK cell frequency with higher expression of activating receptor killer cell immunoglobulin-like receptor (KIR2DS4) and lower expression of inhibitory receptors NKG2A than MRD^–^ patients [28]. As MM progresses, NK cells downregulate activation regulators and upregulate immune checkpoint molecules, such as cytokine-inducible SH2-containing protein (CISH) and TIGIT. Chronic stimulation of NK cells leads to an exhausted phenotype, suggesting that NK cell dysfunction exacerbates with advancing disease [29]. Patients with RRMM and pSCT have upregulated IM3, TIGIT, ICOS, and GITR on NK cells compared to that observed for healthy controls, suggesting exhausted NK cell phenotype; this could be a potential consequence of chronic encounters with the tumor during disease progression.

#### Other markers

NK cells mainly develop in the BM but acquire the ability to migrate to peripheral tissues as they mature, which contributes to their effector function in immune surveillance [30]. Infiltration of cytotoxic NK cells in the tumor microenvironment (TME) is reported to be a prognostic marker for various cancers [31–33]. Furthermore, Ponzetta et al. demonstrated that the number of effector NK cells declines rapidly and selectively in the BM during MM growth in tumor-bearing mice, indicating defective retention of NK cells in the BM during MM [34]. This altered distribution of NK cell subsets is linked to changes in chemokine/chemokine receptor axes in the TME [34]. Levy et al. also demonstrated the role of a CXC chemokine receptor 4 (CXCR4) variant in improved BM homing of NK cells, thereby strengthening the effect of NK cell-based immunotherapies against malignancies [35].

In MM, an imbalance is observed in the proportion of certain chemokine ligands involved in BM localization of NK cells. CXCL9 and CXCL10 (CXCR3 ligands) levels increase, whereas CXCL12 (CXCR4 ligand) levels decrease, mediating exit of resident NK cells from the BM [34, 36]. BM localization of transferred CXCR3-deficient NK cells was reported to be enhanced in healthy and MM-bearing mice. Upregulation of CXCR3 ligands and downregulation of CXCL12 inhibited BM localization of NK cells, weakening their antimyeloma response [34]. The CXCR3/ligand axis is a poor prognostic factor for MM. Higher serum CXCL9, CXCL10, and CXCL11 levels are significantly correlated with lower OS. Moreover, CXCL10 is a better prognostic factor than CXCL9 since the former is not affected by age [36]. MM promotes exit signals, driving effector NK cells outside BM via CXCR3 ligand upregulation and CXCL12 downmodulation, therefore weakening the antitumor immune response at the primary site of tumor growth. Furthermore, inhibition of CXCR3 on NK cells can interfere with the exit of NK cells from the BM [34]. CD38, another surface marker, is a transmembrane glycoprotein that is expressed predominantly on NK cells [37]. CD38 acts as a receptor and has been shown to deliver signals in NK cells, triggering cytotoxic responses in activated human NK cells [38, 39].

Overall, homing of NK cells in the BM may improve MM outcomes, and the CXCR3/ligand axis is the key factor behind induction of NK cell homing signals.

### Functional status of NK cells in MM

Numerous factors within the BM microenvironment have been shown to contribute to NK cell dysfunction. Hypoxia, soluble mediators, such as prostaglandin E2 (PGE2), and inhibitory cytokines, such as transforming growth factor beta (TGF-β) and interleukin (IL)-6, inhibit NK cell activation via various mechanisms [40, 41]. Moreover, based on MM stage, decrease in NK cell function is observed due to expression of tumor-derived immunosuppressive factors, increased tumor burden, and cytokine disturbance.

Progression of MGUS to MM is associated with the loss of activating receptor NKG2D ligand, MICA shedding [24], and upregulation of HLA-1 (an inhibitory KIR ligand) [42]. MM cells express high levels of endoplasmic reticulum protein 5 (ERp5), a protein disulfide isomerase that promotes MICA shedding, which prevents NKG2D binding [43]. Additionally, NK cells can kill via the Fas/Fas ligand (FasL) pathway [44]; however, Fas downregulation and loss-of-function mutations in the gene encoding the Fas antigen are reported in MM, rendering these cells resistant to lysis induced via this pathway [45].

High total serum immunoglobulin level due to M-protein overproduction is often a pathognomonic feature of MM, which could prevent antibody-dependent cell-mediated cytotoxicity (ADCC) exhibited by NK cells [46]. Suppression of NK cell activity and of ADCC has been reported by both high concentration of pooled polyclonal antibodies and purified monoclonal antibodies (mAbs) from a patient with MM [47, 48]. Dose-dependent inhibitory effect of monoclonal IgG on NK cell activity was significantly stronger in patients with active myeloma than in normal controls, but exogenous IL-2 could overcome this effect [49].

In MM, NK cell function is affected by the following factors: changes in the levels of cytokines, soluble factors, and soluble ligands; modulation of NK cell surface receptors; and interference with effector functions, namely ADCC. Dysfunctional NK cells are associated with disease progression and poor prognosis in MM [43], whereas higher NK cell activity improves cumulative survival [50].

NK cells activated with IL-2 and IL-15 are efficient killers of MM cell lines and autologous myeloma cells [51]. MM cells also secrete soluble factors, such as PGE2, that suppress NK cell-mediated antitumor activity. Mesenchymal stromal cells (MSCs) are also an important source of PGE2 in the BM. PGE2 inhibits activating signals transduced by NCR, NKG2D, and CD16, and is actively produced in BM cultures from patients with MM [40]. Abnormal glycosylation, such as hypersialylation, has been shown to play an important role in evading NK cell-mediated immune surveillance in MM [52]. Sialofucosylated structures, particularly Sialyl Lewis a/x-enriched MM cell population, can bind to platelets through P-selectin and protect myeloma cells from NK cell-mediated killing [53]. Thus, disruption of platelet–MM cell interaction using P-selectin-blocking antibodies can be exploited as a therapeutic strategy to enhance NK cell-mediated killing of MM cells [53]. In MM, strong expression of P-selectin glycoprotein ligand-1 (PSGL-1) and sialic acid-derived ligands for Siglec-7 (Siglec-7 L) has been reported. PSGL-1 is highly expressed in primary samples as well as on MM cell lines. Removing sialic acids (desialylation) from MM cell surface or targeted knockout (KO) of Siglec-7 on NK cells enhances NK cell-mediated cytotoxicity [52]. Therefore, targeted desialylation and/or deletion of the gene encoding Siglec-7 can be exploited as a therapeutic strategy to enhance NK cell-mediated killing of MM cells.

### NK cell proportion and fitness in MM

Data describing the overall impact of MM on NK cell development and frequency are limited. Pazina et al. demonstrated that NK cell frequency in PB samples of patients with NDMM and SMM was unchanged compared to that of healthy donors [13]. Conversely, Venglar et al. reported an overall decrease in NK cell frequency, along with accumulation of CD56^bright^ CD16^−^ cytokine-producing subsets, impaired functional properties, and alterations of surface effector receptors during disease progression in patients with MM compared to that in healthy controls [54].

The quantitative profile (number and fitness) of NK cells in MM is different from that under normal homeostatic conditions. High activity and increased number of NK cells correlate with lower tumor burden. A higher number of and more active NK cells exist in PB during early-stage MM (MGUS) than during later stages as disease progresses [55, 56]. CD57^+^CD16^+^ NK cell number is lower in MM than in MGUS. Similarly, IL-10 level is higher in MM and inhibits NK cells, whereas the level of IL-1, which activates NK cell proliferation, is lower in MM [56]. The host immune response of BM NK cells against MM is inhibited via the following events: decreased ADCC, especially in advanced disease, decreased NKG2D, 2B4, and DNAM-1 (without changes in NCR) expression, and *de novo* programmed cell death receptor (PD-1) expression [57]. NK cell proportion has been correlated with MRD status after autologous stem cell transplantation (ASCT), with NK cell frequency being higher in MRD^−^ patients than in MRD^+^ patients [28].

## Effect of MM TME on NK cells

MM modulates the BM environment to create a unique microenvironment favoring uncontrolled tumor proliferation, apoptotic resistance, and antitumor immunity impairment [58]. The MM TME consists of a heterogenous population of MM cells, tissue-resident and infiltrated immune cells, secreted cytokines, MSCs, osteoclasts, osteoblasts, and myeloid and lymphoid cells in a hypoxic environment [58, 59]. Below, we have summarized the roles of MM and MM–TME interaction in MM disease pathology.

### Tumor-induced effect

Chronic tumor-induced stimulation might result in terminally differentiated and exhausted NK cells. A longitudinal study on blood immunophenotyping revealed significantly lower absolute numbers of NK cells in MRD^+^ patients than in MRD^−^ patients, wherein, phenotypically, the NK cells in MRD^+^ patients displayed upregulation of KIR2DS4 (activating receptor) and downregulation of inhibitory molecules NKG2A compared with those in the MRD^−^ group [28]. Furthermore, significantly higher CD94^low^CD56^dim^ NK cell number was reported in PB and BM samples from untreated newly diagnosed (ND) patients with MGUS and SMM. CD94^low^ CD56^dim^ NK cells were the main cytotoxic NK cell subset, and their proportion increased as the disease progressed. Cytotoxicity depended on the contact of NK cells with MM cells via interaction with CD56–DNAM-1 present on MM cells. The accumulation of CD94^low^CD56^dim^ NK cell subset in the early phase of disease provides rationale for employing NK cell-based therapeutic strategies in early stages of MM and highlights the importance of strategies supporting expansion and infusion of CD94/NKG2A^−^ NK cells [26]. DNAM-1 activating receptor is essential for proper activation and accumulation of CD94^low^CD56^dim^ NK cells by MM cells. Reduced DNAM-1 expression has been reported on CD56^dim^ NK cells from patients with active disease compared to that from patients in remission or healthy controls [26]. Low to undetectable HLA-G transcript levels have been reported in MM cells [60]. High HLA-I and low MICA levels have been reported in late-stage pleural effusion cells (resistant to NK cells) from patients with MM as opposed to that in early-stage MM cells in the BM, which were readily recognized by NK cells [61]. NKG2D ligand-expressing MM cells cause significant decrease in NKG2D^+^ NK cell number because of NKG2D downregulation caused by persistent contact with NKG2D ligands expressed on MM cells [24]. Impaired activating receptor expression on NK cells provides the potential mechanism of innate immune escape. Substantial downregulation of three major NK cell activating receptors, namely NCR3/NKp30, NKG2D, and 2B4/CD244/p38, in the BM has been reported in patients with MGUS/MM [25]. Moreover, normal NCR and NKG2D expression but significantly weaker expression of coreceptor 2B4/CD244 and low-affinity Ig-Fc receptor CD16 is reported in patients with MM [42]. SUMOylation of activating receptor DNAM-1 ligand PVR in MM cells prevents its expression on MM cells, impairing DNAM-1-mediated NK cell recognition [62].

Thus, chronic tumor-induced NK cell activation during disease progression has been reported to have profound effects on NK cell function, with downregulated activating signals and upregulated inhibitory signals impacting response outcome.

### Cytokine-induced effect

Cytokines IL-6 and IL-10 impair NK cell activity and decrease disease-free survival, respectively [7]. Although TNF-α contributes to MM pathology by generating malignant plasma cells, *TNF-α* promoter polymorphism, especially *TNF-α* -238 GA + AA genotype, is associated with significant improvement in PFS and OS in MM, whereas *TNF-α* -308 GA and GA + AA genotypes are associated with reduced risk of MM, indicating a role of *TNF-α* promoter polymorphism in MM pathology. These polymorphisms may mediate multiple functions that need further investigation [63]. TGF-β inhibits CD16-mediated NK cell IFN-γ production and ADCC mediated by SMAD family member 3 (SMAD3) [64].

### Immune cell-induced effect

The MM TME is immunosuppressive due to high levels of IL-10, TGF-β, IL-4, regulatory T cells (T_regs_, MDSCs, and reduced cytotoxic T lymphocyte number, resulting in suppressed humoral and cytotoxic immunity [59]. T_regs_ are exploited by the tumor/TME to limit antitumor immunity. Protumor outcomes of T_regs_ are based on the release of inhibitory cytokines IL-10, IL-35, and TGF-β; cytolysis: granzyme A- and B-dependent and PRF1-dependent killing of NK and T cells; and metabolic disruptions: high affinity IL-2 receptor α (CD25)-dependent cytokine deprivation-mediated apoptosis, cyclic adenosine monophosphate mediated inhibition, CD39- and/or CD73-generated immunosuppression, adenosine-purinergic adenosine (A2A) receptor-mediated immunosuppression, and modulation of dendritic cell (DC) maturation. Moreover, MDSCs are negative prognostic markers in MM [41], and BM MSCs mediate immune resistance in MM [16, 65].

Thus, T_regs_, MDSCs, and BM MSCs are major immune cell-mediated barriers for NK cell activity in the MM TME.

### Hypoxia and adenosine-induced effect

Hypoxia abolishes the killing potential of NK cells isolated from healthy donors (HD) via downregulation of surface activating receptors [66]. Moreover, hypoxia-induced autophagy in malignant cells causes granzyme B degradation, rendering NK cells less cytotoxic in the hypoxic TME [67]; decrease in TME hypoxia decreases intracellular PRF1 levels without any changes in the surface expression of NK cell ligands (HLA-A/B/C/E, MICA/B, and ULBP1/2) and receptors (KIR, NKG2A/C, DNAM-1, NCRs, and 2B4) [66]. Furthermore, the metabolism and expression of activating receptors on NK cells are influenced by adenosine nucleotidase CD73 overexpression, causing accumulation of extracellular adenosine; this exerts immune metabolic effects on NK cells in the TME. Adenosine stimulation of A2A receptors on NK cells negatively affects NK cell metabolism, cytokine production, and cytotoxicity [68]. Adenosine is a potential marker of myeloma progression, and its level increases with disease progression [68].

Overall, hypoxia and adenosine in the BM microenvironment render NK cells less cytotoxic, and NK cell survival is inversely associated with the activation of HIF-1α and adenosine A2A receptors. This provides a potential solution for overcoming immunosuppressive effect of adenosine on NK cells by blocking CD39/CD73 and CD38 [68, 69].

## Effect of approved antimyeloma therapies on NK cells

**Fig. 2:**
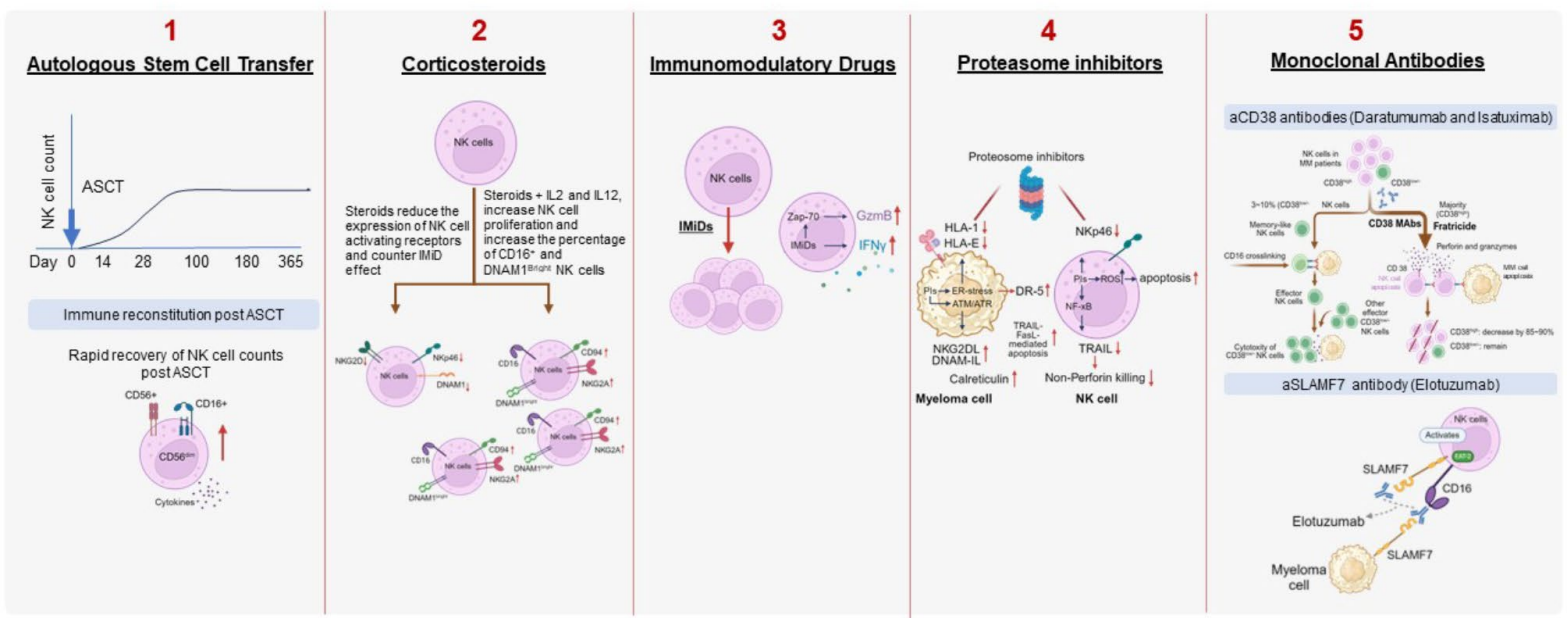
Effect of antimyeloma therapy on NK cells. **1. Autologous stem cell transfer**. NK cells rapidly reconstitute after ASCT. Post-ASCT, NK cells show an increase in proliferative capacity and phenotypic redistribution, resulting in an increase in CD56^dim^ CD16^+^ NK cell frequency (adapted from *Trans and Cell Ther 28 (2022) 310.e1-310.e6)* [70]. **2. Corticosteroids**. Corticosteroids (dexamethasone) inhibit the development, proliferation, and function of NK cells in MM. Concomitant use of dexamethasone with lenalidomide inhibits the immunomodulatory effects (NK cell activating ability) of lenalidomide and also downregulate NKG2D, NKp46 and DNAM1. However, dexamethasone increases NK cell proliferation, CD16^+^ and DNAM1^bright^ NK cell numbers, CD94 or NKG2A expression in the presence of IL2 and IL12. **3. Immunomodulatory drugs**. IMiDs increase the activation and proliferation of NK cells (CD56^dim^ NK cells increase) as well as repair of lytic synapse. Apart from the antimyeloma effect, IMiDs produce costimulatory effects on NK cells, resulting in increased NK cell number. IMiDs also enhance NK cell cytotoxicity by ZAP-70-mediated CD56^dim^ NK cell increase, and upregulation of GZM-B, and IFNγ. **4. Proteasome inhibitors**. PIs sensitize NK cells against myeloma cells by upregulation NKG2 ligands and DNAM1-L. Calreticulin, an NKp46 ligand, can be externalized by the induction of ER stress pathway. ER stress also induces DR5 expression and enhances TRAIL-mediated killing of myeloma cells. Bortezomib causes apoptosis of primary resting NK cells by inducing ROS production and reducing NKp46 receptor expression. as well as NK cell cytotoxicity-mediated by the NKp46 activation (adapted from *Front. Cell Dev. Biol. 12:1359084*) [71]. **5. Monoclonal antibodies**. CD38 monoclonal antibodies (daratumumab and isatuximab) cause fratricide of CD38^high^ NK-cells. Majority of NK cells (85 ~ 90%) express CD38 and are targeted by CD38 antibodies. The residual CD38^low/−^ NK cell population (3–10%) are resistant to fratricide and show high proliferative and cytotoxic activity. The effector CD38^low/−^ NK cells show a high proliferative potential and functional activity in the presence of CD38 mAb by inducing ADCC. However, the NK cells recover once the treatment is discontinued (adapted from *Int J Bio Sci 18* [5]:*1974–1988)* [72]. SLAMF7 targeting monoclonal antibodies (elotuzumab) acts by antibody dependent cellular cytotoxicity. SLAMF7 is also expressed on NK cells, where it acts as an activating receptor via interaction with signaling adaptor protein, EWS-activated transcript 2 (adapted from *Clin Transl Sci (2018) 11*,* 261–266)* [73]. **ADCC**, antibody-dependent cellular cytotoxicity; **ASCT**, autologous stem cell transfer; **ATM/ATR**, ataxia-telangiectasia mutated/RAD3-related; **CD**, cluster of differentiation; **DNAM1**, DNAX accessory molecule; **DR**, death receptor; **EAT2**, EWS-activated transcript 2; **ER**, endoplasmic reticulum; **FasL**, Fas ligand; **GZM-B**, granzyme B; **HLA**, human leukocyte antigen; **IFN-γ**, interferon gamma; IL, interleukin; **IMiD**, immunomodulatory drugs; **MM**, multiple myeloma; **NK**, natural killer; **PI**, proteasome inhibitor; **ROS**, reactive oxygen species; **SLAMF7**, signaling lymphocyte activation molecule family member 7; **TRAIL**, tumor necrosis factor related apoptosis-inducing ligand; **ZAP-70**, Zeta-chain-associated protein kinase 70

Although MM outcomes have improved due to the use of the available treatments, their effect on the cytotoxic ability of NK cells in MM is unclear. This section describes the pharmacodynamic effects of these treatments on NK cells (Fig. 2) along with their shortcomings and future perspectives (Table 1).

**Table 1:** Effect of current MM treatments on NK cell cytotoxicity

Study	Treatment	Effect on NK cells	Mechanism
*A. Autologous Stem Cell Transplant*			
Parrado et al. [74]	**ASCT**	• NK cells (CD56 + and CD16 + subsets) recover faster than T and B cell subsets o However, the CD3-CD56+, CD3-CD16+, CD16 + CD56+, CD3-CD8+, and especially the CD3CD57+, CD16 + CD57+, and CD56 + CD57 + subsets had a slower recovery than the global CD56+, CD16+, or CD57 + subsets	Not available
Reuff et al. [75]	**ASCT**	• CD8 T cell and NK cell counts recovered rapidly to pre-transplantation levels o Patients with high NK cell count (> 200/µL) at 1 month after HSCT were associated with significantly prolonged PFS versus patients with lower NK cell count (< 100/µL)	Not available
Orrantia et al. [76]	**ASCT**	• Phenotypic redistribution during reconstitution after ASCT such as ↑proliferative capacity and immature phenotype • ↓in CD56^Dim^ and ↑in CD56^Bright^ NK cell frequency • Degree of NK cell maturation after ASCT affects the clinical outcome o ↓frequencies (versus ↑frequencies) of mature highly differentiated NKG2A-CD57 + NK cell after 30 and 100 days of ASCT correlated to superior PFS and longer time to next treatment	• At Day 13, ↑proliferative capacity of immature phenotype (↓in CD56^Dim^ and ↑in CD56^Bright^ NK cell frequency); however, attains pre-transplant levels at Day 30 • At Day 30, complete recovery of NK cells • After 30 and 100 days of ASCT, ↓frequencies (versus ↑frequencies) of mature highly differentiated NKG2A-CD57 + NK cell correlated to superior PFS and longer time to next treatment
Keruakous et al. [77]	**ASCT**	• NK cell count was significantly ↑in the MRD-negative patients vs. MRD positive • High absolute NK cell counts 2–3 months after ASCT is an independent predictor for MRD negativity in MM for patients treated with ASCT	Not available
D’Souza C et al. [29]	**ASCT**	• There was no difference in CD16 + NK cells before and after induction treatment, but there were less mature CD57 + NK cells after ASCT	Not available
*B. Dexamethasone*			
Morgan et al. [78]	**DEX**	• **DEX** exerts dichotomic effect o NK cell effector response is initially ↓in absence of favorable local cytokine milieu • ↑the NK cell proliferation, survival, and reactivity in a secondary recall response in the presence of cytokines present	• DEX in presence of IL-2 and IL-12: o ↑ NK cell proliferation o ↑ CD16 + and DNAM1^Bright^ NK cells percentage o ↑ Expression of CD94 or NKG2A o ↑ Mitochondrial function of NK cells ↑ IFN-γ response
Chen et al. [79]	**DEX**	• **DEX** ↓ the proportion of NK cells in spleen along with ↓their function	• ↓ Ly49G and NKG2D receptors • May shift NK cell subpopulations toward an immature stage ↑ CD11b-CD27 + and ↓ CD11b + CD27-
Gandhi et al. [80]	**DEX + LEN**	• In **LEN + DEX** combination, **DEX** ↑the antiproliferative effects of **LEN** but ↓its immunomodulatory effects i.e., NK cell activating ability of lenalidomide in a dose dependent manner (as measured by IFN-γ and GZM production)	Not available
Carter et al. [81]	**DEX + LEN**	• **LEN + DEX** combination is unable to exert its positive immunomodulatory effects on the NK cell NCR receptors	• Treatment-dependent ↓in NK cell receptor expression although individual receptors were affected independently
*C. Immunomodulatory Drugs*			
Davies et al. [82]	**THAL**	• Mediates anti-MM cytotoxic effects	• Modulate NK cell proportion and function as indicated by o ↑ CD3-CD56 + cells o MM cytotoxicity independent of MHC o Blocked the MM cytotoxicity upon CD56 + cell depletion
Fionda et al. [83]	**LEN**	• **LEN** ↑ NK cell activating ligand expression	• ↑ CRBN-mediated degradation of repressors (transcription factors IKZF-1/IKZF-3 and IRF4) of NK cell activating receptors MICA and PVR/CD155
Hideshima et al. [84]	**LEN** **POM** **Iberdomide**	• **LEN** and POM↑ NK and T cell cytotoxicity • **Iberdomide** ↑NK cell activity in dose dependent manner	• ZAP-70-mediated CRBN independent mechanism of LEN o ↑ GZM B expression by directly binding to ZAP-70 transphosphorylation • CRBN-mediated ZAP-70 independent o CRBN-mediated IKZF3 degradation • **Iberdomide** induced ZAP-70, upregulated GZM B, and consequently downregulated IKZF3
*D. Proteasome Inhibitors*			
Wang et al. [85]	**BOR**	• **BOR** may ↓rNK cell-mediated immunity	• Significant rNK cell apoptosis, possibly due to ROS accumulation, in dose and time dependent manner • ↓NKp46 receptor expression in dose dependent manner; levels of DNAM-1, NKG2D, NKP30, and perforin were unaffected
Yang et al. [86]	**CAR**	• **CAR** ↑NK cell degranulation and MM cell line sensitivity to NK cell-mediated lysis (via HLA and TRAIL pathway)	• ↑degranulation o Percentage of CD107a + NK cells was substantially ↑after treatment with **CAR** • ↑MM cell lysis o ↓expression of HLA-I in (time and dose dependent) and ↓newly formed HLA-I o ↑of DR4 and DR5 expression on MM cells, which could ↑NK cell killing via TRAIL pathway. However, blocking TRAIL did not affect NK cell cytotoxicity • No effect on MICA A/B, ULBP 1–3, NKp30-L, NKp44-L, and NKp46-L, and blocking NKG2D and NCRs on NK cells did not affect NK cell cytotoxicity
Niu et al. [87]	**BOR + iNK cells/** **γδ T cells**	• **LD-BOR + iNK cells** or **γδ T cells** provide synergistic cytotoxic effect on MM cells	• **LD-BOR** ↑the expression of NKG2D and DNAM-1 ligands on MM cells, which sensitized the MM cells to lysis by induced NK and γδ T cells
Carlsten et al. [88]	**BOR + eNK cells**	• NKG2ASp NK cell cytotoxicity significantly ↑on **BOR** pretreated MM (versus. untreated)	• NK cell sensitization via NKG2A/HLA-E axis: ↓of MM cell surface expression of HLA-E, which was independent of **BOR’s** previously reported NK cell mediated MM cytotoxic mechanism (↑of the TRAIL death receptor DR5 on the surface of MM cells)
*E. Monoclonal Antibodies (Anti-CD38 and Anti-SLAMF7)*			
Casneuf et al. [89]	**DARA**	• After first **DARA** dose, total and activated NK cell counts ↓rapidly and remained low during treatment and recovered after treatment ended. NK cells may still contribute to ADCC since the NK cell counts were not completely depleted and retained cytotoxic functionality • Observed maximum effect relationship between **DARA** dose and NK cell reduction	• DARA induced ADCC by fratricide of NK cells
Wang et al. [90]	**DARA + eNK cells**	• CD38 + NK cells were almost eliminated; CD38-/low NK cells survived • CD38^-/low^ NK cells were more effective in eradicating MM cells than were CD38 + NK cells in the presence of **DARA**	• CD38 + NK cell depletion occurred due to daratumumab-induced NK cell fratricide via antibody-dependent cellular cytotoxicity (ADCC) • CD38−/low NK cells displayed a significantly enhanced potential for expansion than CD38 + NK cells. Expanded NK cells derived from the former population exhibited higher cytotoxicity
Kararoudi et al. [91]	**DARA**	• Adoptive immunotherapy using ex vivo expanded CD38 knockout NK cells have the potential to boost **DARA** activity in MM o CD38 knockout NK cells were resistant to **DARA**-induced fratricide, showed enhanced persistence, and ↑ADCC activity against CD38-expressing MM cell lines and primary MM cells • In addition, transcriptomic and cellular metabolic analysis demonstrated that CD38 knock-out NK cells have unique metabolic reprogramming with ↑mitochondrial respiratory capacity	• Resistance to **DARA** induced ADCC by fratricide of NK cells
Collins et al. [92]	**ELO**	• **ELO** ↑NK cell cytotoxicity against SLAMF7 + MM cells, but not against SLAMF7 + NK cells	• Binding to NK cell CS1 • ELO may also ↑CS1–CS1 interactions between NK cells and CS1(+) target cells to ↑cytotoxicity in an ADCC-independent manner. NK cell activation may depend on differential expression of EAT-2, which is present in NK cells but absent in primary, human MM cells
Pazina et al. [93]	**ELO**	• Strongly ↑degranulation and activation of NK cells in a CD16-dependent manner • A non-fucosylated form of **ELO** with higher affinity for CD16 exhibited ↑potency	• CD16-dependent stimulation of NK cells o **ELO** can induce trans-costimulatory signals upon direct engagement with SLAMF7
		• Potential to ↓activation thresholds of other NK cell receptors engaging with MM cells	• CD-16 independent trans-costimulation through SLAMF7 direct and indirect activation o Direct ligation with SLAMF7 on NK cells o Direct engagement of **ELO** with SLAMF7 on NK cells can initiate intracellular calcium signaling responses triggered by multimeric engagement of NKp46 and NKG2D

**ADCC**, antibody dependent cell cytotoxicity; **ASCT**, autologous stem cell transplantation; **BOR**, bortezomib; **CAR**, carfilzomib; **CD**, cluster of differentiation; **CR**, complete remission; **CRBN**, cereblon; **CS1**, chorismate synthase 1; **DARA**, daratumumab; **DEX**, dexamethasone; **DR5**, death receptor 5; **EAT-2**, EWS-activated transcript 2; **ELO**, elotuzumab; **eNK cell**, endometrial NK cell; **FasL**, Fas ligand; **GM-CSF**, granulocyte-macrophage colony-stimulating factor; **GZM**, granzyme; **HLA**, human leukocyte antigen; **HSCT**, hematopoietic stem cell transplantation; **IFN-γ**, interferon-γ; **IKZF1**, Ikaros; **IKZF3**, Aiolos; **IL**, interleukin; **iNK cell**, induced NK cell; **IRF4**, interferon regulatory factor 4; **ISA**, isatuximab; **LD**, low-dose; **LD-CYC**, low-dose cyclophosphamide; **LEN**, lenalidomide; **MHC**, major histocompatibility complex; **MM**, multiple myeloma; **MIC**, MHC class I chain-related genes; **MIP**, Macrophage inflammatory protein; **MRD**, minimal residual disease; **NCR**, natural cytotoxicity receptor; **NK**, natural killer cell; **PBMC**, peripheral blood mononuclear cell; **PFS**, progression free survival; **PRED**, predinisone; **PVR**, poliovirus receptor; **ROS**, reactive oxygen species; **rNK cell**, resting NK cell; **SLAMF**, signaling lymphocyte activation molecular family; **THAL**, Thalidomide; **TNF**, tumor necrosis factor; **TRAIL**, TNF related apoptosis inducing ligand; **ULBP1**, UL16 binding protein 1; **ZAP-70**, zeta-chain-associated protein kinase 70

### ASCT

NK cells are the first lymphoid cell type to reconstitute after ASCT [74]. Post ASCT, the proliferative capacity and phenotypic redistribution of NK cells increase, resulting in a decrease in mature CD57^+^ and CD56^bright^ NK cell frequency and an increase in CD56^dim^ NK cell frequency [29, 76]. Moreover, specific subsets of CD56^bright^ cells, such as CD3^–^CD56^+^ and CD16^+^CD56^+^ cells, exhibit slower recovery than global CD56^bright^ NK cells [74]. Similar pattern was observed for CD16^+^ and CD57^+^ NK cell subsets [74]. The impact of adaptive post-ASCT NK cell expansion reveals that patients with higher absolute adaptive NK cell numbers (> 1.58/µL) at day 28 (post ASCT) were associated with significantly decreased relapse risk than patients with lower numbers [76].

In MM, improvement in NK cell proportions after ASCT produces favorable PFS, relapse rate, MRD negativity, and time to next treatment outcomes. However, not all phenotypes and subsets of NK cells are equally efficacious post-transplant [76, 77]. Recently, adaptive NK cells have emerged as a key phenotype. Adaptive NK cells mediate protective memory responses following cytomegalovirus (CMV) infection, and they express the maturation marker CD57 and the activating receptor NKG2C. In CMV-seropositive individuals who underwent ASCT for MM, expansion of adaptive NK cells on day 28 was associated with more than double the time to relapse than that for patients without adaptive NK cell expansion [70]. CMV infection helps in the expansion of NKG2C^+^/NKG2A^−^ NK cells [94]. Moreover, based on the 28-day values, early recovery of adaptive NK cells or tumor-specific NK cells could mediate superior outcomes after ASCT [76]. IL-15 influences NK cell recovery and clinical outcome after ASCT. Rapid post-transplant NK cell recovery was reported to peak at 1 month and decline to base level in the first-year post-transplant; IL-15 plasma levels peaked at the day of the transplant and declined sharply afterward, suggesting rapid consumption of IL-15 by cytokine-consuming NK cells. Elevated IL-15 levels contribute to antitumor efficacy by inducing the recovery of NK subsets and by modulating the expression of activating receptors, NKG2D, NKp30, and NKp46 [95]. Post-ASCT NK cell redistribution and phenotypic changes were observed in MM [76]. NK cells undergo phenotypic changes characterized by increased proliferation and immature phenotype. Lower frequencies of the terminally differentiated NKG2A^−^CD57^+^ NK cells at > 30 and > 100 days post-auto HSCT were associated with better PFS [76]. Addition of anti-CD38 mAbs to the triplet backbone yields unprecedented depth of response and MRD negativity in NDMM [96–98]. Longitudinal immune profiling of PB and BM cells derived from a cohort of patients with NDMM prospectively treated with quadruplet induction and ASCT revealed that quadruplet induction negatively affected the NK cell compartment during induction, and NK cells did not completely recover in the immediate post-transplant period [99].

Hence, enhanced recovery rate of a specific subset of NK cells can be leveraged to improve MM outcomes post ASCT.

### Steroids

Dexamethasone is a key adjunct in most treatment regimens across all phases of MM. It inhibits the development, proliferation, and function of NK cells in MM [79]. Although dexamethasone enhances the antiproliferative effects of lenalidomide in MM cells, it inhibits the immunomodulatory effects (NK cell activating ability) of lenalidomide in a dose-dependent manner, when administered concomitantly [80, 81]. Dexamethasone reduces NK cell activity by inducing treatment-dependent reduction in DNAM, NKG2D, and NKp46 receptor expression and counters the immunomodulatory effect of lenalidomide [81]. However, in the presence of IL-2 and IL-12, dexamethasone increases NK cell proliferation, CD16^+^ and DNAM1^bright^ NK cell numbers, and CD94 or NKG2A expression, and improves their mitochondrial function [78]. Paiva et al. studied the effect of steroids on T lymphocytes and NK cells of high-risk patients with SMM included in the QUIREDEX trial. To determine whether corticosteroids exert a detrimental effect on the immunomodulatory activity of lenalidomide, longitudinal samples were analyzed at baseline and after 9 cycles of dexamethasone/lenalidomide administration; CD56^dim^ cytotoxic and CD56^bright^ immune regulatory NK cells were significantly activated after therapy, and lenalidomide modulated the proliferation rate of immune cells of high-risk patients with SMM in the presence of dexamethasone [100].

Overall, NK cell activity in MM during dexamethasone therapy may depend on the cytokine milieu. Hence, longitudinal cytokine profile (before and after dexamethasone therapy) should be evaluated to assess NK cell activity. Moreover, since concomitant dexamethasone and lenalidomide administration exerts antiproliferative effects on MM [80], a combination of low-dose dexamethasone and lenalidomide should be investigated to assess whether NK cell activity can be preserved while maintaining the antiproliferative effect of the combination.

### Immunomodulatory drugs

Immunomodulatory drugs (IMiDs; e.g., lenalidomide and pomalidomide) are important standard-of-care agents for post-transplant maintenance of MM, NDMM, or RRMM [101]. IMiDs have costimulatory effects on NK cells apart from pleiotropic antimyeloma properties and antiproliferative effects [82]. Immunomodulatory drugs activate T cells and enhance NK cell cytotoxicity mainly by ZAP-70-mediated cereblon (CRBN)-independent and CRBN-mediated ZAP-70-independent pathways [84].

IMiDs have been successfully combined with dexamethasone for treating MM, but the combined effects of lenalidomide and dexamethasone on NK cells remain to be elucidated. A recent study revealed that this combination could not repair the exhaustion of NK cells in MM [29]. Next generation IMiDs like CC-220 (iberdomide) also enhanced NK cell activity and proliferation in a dose-dependent manner. Iberdomide upregulated granzyme B and consequently downregulated IKAROS family zinc finger 3 (IKZF3); it was more potent than lenalidomide and pomalidomide in improving NK cell activity [84, 102].

Thus, IMiDs have multiple activities, and in combination with other antimyeloma agents, they have proven to be efficacious in augmenting NK cell activity.

### Proteasome inhibitors (PIs)

PIs can sensitize myeloma cells to killing by NK cells via several ways. Upregulation of ligands for NK cell activating receptors (ULBP proteins, MICA/MICB, poliovirus receptor [PVR/CD155]) increases NK cell activation. Calreticulin, which is externalized by bortezomib treatment, has recently been reported to be a ligand for NKp46.

The PI bortezomib causes resting NK cell apoptosis due to accumulation of reactive oxygen species and by suppressing NKp46 expression [85]. In contrast, bortezomib may improve recognition and activation of NK cells by inducing immunogenic cell death (ICD) of MM cells and by stimulating the cGAS/STING pathway [103]. Bortezomib and carfilzomib can improve MM cell cytotoxicity via NK cells alone or in combination with stimulated, induced, or expanded NK cells. Reported mechanisms include (i) decreased HLA-I [86] and HLA-E [88] expression on MM cell membrane; (ii) TRAIL- and FasL-mediated apoptosis resulting from proteasome-induced increased DR4, DR5, and FAS expression on MM cells [86, 88]; (iii) enhanced NK cell degranulation [86]; and (iv) bortezomib-induced NKG2D and DNAM-1 expression on MM cells, wherein DNAM-1 (CD226) expression is required for mounting optimal immune response against MM cells [87]. PIs induce ER stress [104] that translocates calreticulin from the ER to the cell membrane. Externalized calreticulin is recognized by the NK activating receptor NKp46 [105].

Overall, PIs can improve the antimyeloma effect of NK cells by modulating the expression of NK cell receptors and ligands. Thus, optimum scheduling of PI- and NK cell-based combination therapy should be investigated in future, including the hypothesis that NK cell-based treatments should be followed by PI treatments.

### Monoclonal antibodies (mAbs)

#### Anti-CD38

NK cells are key effector cells for mAbs. CD38 is expressed on both NK cells (constitutively) and MM cells [72]. However, shortly after their introduction in clinical setting, it was apparent that CD38 antibodies induce significant fratricide leading to prolonged depletion of NK cells that can last up to 6 months [89].

In NK cells, CD38 activation with agonistic antibodies causes significant increase in intracellular Ca^2+^ levels; releases IFN-γ and granulocyte macrophage colony stimulating factor; and phosphorylates tyrosine residue of CD3-ζ, FcεRIγ chains, zeta-associated protein (ZAP)-70, and casitas B-lineage lymphoma (c-Cbl), thus inducing cytolytic effector functions [39]. Anti-CD38 mAbs, such as daratumumab and isatuximab, induce MM cell death through CD38 binding; this CD38-mediated ADCC also results in the elimination of CD38^+^ NK cells (NK cell fratricide) [72, 90] by leaving an activated CD38^low/−^ NK cell population. However, the NK cells recover once the treatment is discontinued [89].

There is growing evidence that the efficacy of daratumumab is influenced by the proportion of activated NK cells that express CD16 at baseline. In a study, after daratumumab monotherapy, lower proportion of CD16-expressing NK cells with increased expression of TIM3 and HLA-DR (exhaustion markers) were associated with inferior response, PFS, and OS. The development of daratumumab resistance was also associated with an increased percentage of CD56^bright^ BM resident NK cells, which expressed inhibitory receptors and lacked expression of CD16 [106]. KO of CD38 can overcome the problem of fratricide and can enhance the metabolism of NK cells, resulting in enhanced cytotoxicity and persistence. There is a strong argument to combine adoptively transferred allogeneic CD38-KO NK cells with anti-CD38 antibodies, potentially in combination with strategies to induce CD38 expression on MM cells, thus overcome potential resistance to anti-CD38 antibodies [91]. Moreover, with recent advancements, another innovative off-the-shelf strategy for the effective treatment of MM has been developed. It involves engineered CD38-KO NK cells with chimeric antigen receptor (CAR) specific for B cell maturation antigen (BCMA); interleukin-15 fusion; and a high affinity, non-cleavable CD16 [107].

Notably, residual NK cells retain ADCC mediated through CD16a (FcγRIIIA); CD16 engagement on NK-cells triggers Ca^2+^ flux, tyrosine phosphorylation of ZAP-70, mitogen-activated protein kinase (MAPK) activation, and IFN-γ secretion, causing ADCC-mediated target cell death [108]. Although anti-CD38 therapy depletes NK cell number, the CD38^low/−^ NK cell number is sufficient to drive the efficacy of anti-CD38 antibodies; a recent report suggests that the persisting anti-CD38-treated NK cells exhibit an exhausted phenotype characterized by low expression of CD16 and granzyme B and increased expression of TIM-3 and HLA-DR and are associated with both primary and acquired resistance against anti-CD38 antibodies [106].

#### Anti-SLAMF7

An anti-SLAMF7 agent, elotuzumab, induces myeloma cell death via NK cell-mediated ADCC, which is enhanced in combination with lenalidomide. CS1 (or SLAMF7 [a CD2-like receptor-activating cytotoxic cells], CRACC, or CD319) is a type I membrane glycoprotein; it is highly expressed on malignant plasma cells, but its function on MM cells remains unclear [109]. Elotuzumab, a humanized IgG1 mAb that targets SLAMF7 via ADCC and FcγRIIIA engagement (CD16) on NK cells, is approved in combination with dexamethasone and lenalidomide or pomalidomide for MM treatment [109]. SLAMF7 is also expressed on NK cells, where it acts as an activating receptor by interacting with the signaling adaptor protein—EWS-activated transcript 2 (EAT-2) [109]. Elotuzumab enhances NK cell cytotoxicity against SLAMF7^+^ MM cells, but not against SLAMF7^+^ NK cells [92]. Elotuzumab directly binds to SLAMF7 on NK cells, and unlike those on MM cells, the SLAMF7 receptors on NK cells mediate EAT-2-dependent activation of phospholipase C gamma 1 (PLCγ) and extracellular regulated MAP kinase (ERK), and intracellular Ca^2+^ mobilization [92, 93]. Moreover, direct engagement of elotuzumab with SLAMF7 on NK cells potentiates intracellular Ca^2+^ signaling via antibody-mediated aggregation of NKp46 and/or NKG2D [93].

Overall, the antiCD38 mAbs may decrease NK cell activity over a short-term, but NK cells recover once the therapy is discontinued. Although antiSLAMF7 mAbs also interact with SLAMF-7 receptors on NK cells, they exert an overall positive effect on NK cell activity while simultaneously killing MM cells via ADCC.

## Utilizing NK cell function for MM treatment

NK cell-based therapies are emerging as promising antimyeloma treatment option. To fully exploit the potential of NK cells and NK cell-based therapies, existing challenges should be overcome, and NK cell therapy in combination with antimyeloma agents should be investigated. A few strategies to harness the potential of NK cells in MM are mentioned below.

### IMiDs and PIs

As discussed previously, IMiDs are thalidomide analogues, which act via multiple antimyeloma mechanisms and improve NK cell cytotoxicity and proliferation [110]. Additionally, IMiDs have costimulatory effects on NK cells via CRBN-dependent or -independent mechanisms and release of IL-2 and IFN-γ. IMiDs also enhance expression of CD38 and interferon-stimulated genes on myeloma cells through the degradation of two transcription factors, namely Ikaros and Aiolos, that are vital for B-cell lymphogenesis and production of high-affinity BM plasma cells, respectively; thus, IMiDs could be combined with anti-CD38 mAbs to ensure optimal CD38 expression on CD38^dim^ or adoptive cell therapy with CD38-KO NK cells could be deployed [111]. Moreover, a recent study showed that Ikaros and Aiolos play an important role in regulating AP-1 transcriptional complexes and NK development. In addition, the intracellular checkpoint, CISH, is negatively regulated by Ikaros, but CISH expression increases in Ikaros-null NK cells. Therefore, long-term treatment with IMiDs could have some unanticipated negative consequences on NK cell function [112].

Overall, these insights provide a framework for developing novel therapeutic combinations of IMiDs with NK cells and NK cell-based therapies.

Despite the potential negative impact of PIs on NK cells, they can be used with NK cell-based therapies. For e.g., bortezomib treatment upregulates TRAIL DR5 and downregulates HLA-E on the surface of MM cells, resulting in increased MM cell killing by NK cells [88].

Overall, PIs potentiate the antimyeloma activity of NK cells, including ex vivo-expanded NK cells. Carlsten et al. hypothesized that the infusion of ex vivo-expanded NK cells following treatment with bortezomib could eradicate MM cells that would normally evade killing by PIs alone, potentially improving long-term survival among patients with MM [88]. However, this strategy requires optimum scheduling, and NK cell-based therapy could be followed by PIs.

### Checkpoint inhibition

MM cells employ multiple strategies for immune evasion; expression of inhibitory receptors and immune checkpoint proteins are reported to be crucial for myeloma immune evasion, wherein MHC-I expression is crucial [44, 61]. Strategies to overcome this limitation have focused on the blockade of NK cell inhibitory receptors via mAbs. IPH2101, a human antiKIR2D mAb, enhances NK-mediated killing of autologous MM cells, albeit without substantial monotherapy success in clinical trials [113]. Another strategy is to prevent HLA-E-mediated NK cell suppression by blocking NKG2A engagement. Various trials are investigating the NKG2A inhibitory receptor-blocking mAb—monalizumab—in different malignancies, including MM [114]. Higher frequency of TIM3^+^ and HLA-DR^+^ NK cells are associated with anti-CD38 antibody resistance [106]. Anti-TIM3 antibodies may overcome this resistance and provide a rationale for the anti-TIM3 and anti-CD38 antibody combination therapy. TIGIT could also be involved in weakening NK cell response to MM. Preclinical models of anti-TIGIT mAbs exhibit enhanced NK cell antitumor activity. Impressive preclinical results have been observed with anti-KIR2D mAbs, anti-NKG2A antibodies, PD-1/PD-L1 immune checkpoint inhibitors, and anti-TIGIT antibodies, but the success of preclinical results has not yet been replicated in clinical trials [7].

### NK cell redirection and NK cell adoptive therapeutics

Other than mAbs, several NK cell engagers (NKCEs) are being introduced in clinical settings, namely bispecific killer cell engagers (BiKEs) and trispecific killer engagers (TRiKEs). NKCEs simultaneously bind to the tumor target antigen(s) and activate receptor(s) on endogenous NK cells to form a biological synapse [7, 115]. BCMA has emerged as a promising target for MM treatment due to its almost universal expression on tumor cells and limited expression in other tissues. Preclinical data on AFM26, a novel BCMA-directed tetravalent bispecific antibody, revealed its antimyeloma effects [116]. A BCMA/CD16a bispecific NKCE, RO7297089, showed important preclinical activity, but resulted in modest clinical activity as a single agent [117]. CTX-8573, an anti-BCMA IgG1 antibody, comprises antiNKp30 Fab fragments and afucosylated Fc for enhanced CD16a engagement. CTX-8573 showed potent tumor cell killing by NK cells in preclinical models [118]. SAR445514, an anti-BCMA NKCE, co-engages NKp46 and CD16a on NK cells and thus redirects activated NK cells to kill BCMA^+^ tumor cells; it is in Phase 1 development stage (NCT05839626) [119].

NKCEs have the following advantages over CAR-NK cells: antitumor action can be strengthened by binding to > 1 activating receptor; absence of vector-mediated transfer and simpler manufacturing process; fusion of IL-2 or IL-15 allowing NK cell expansion; low cytokine induction; and different formats, e.g., IgG-based format with longer half-life or smaller IgG fragment [7, 115]. A challenge associated with NKCE therapy is that its efficacy is dependent on the functionality of the NK cells of patients, which may be impaired due to prior therapy or BM microenvironment. Although NK cells may retain ADCC after anti-CD38 antibody therapy, their depletion could affect ADCC in MM [7]. Therefore, efforts focused toward shielding NK cell depletion are of high interest [7], and one such strategy is allogeneic CD38-KO NK cells [75]. Since CD38^−/low^ NK cells display better potential for expansion and are more cytotoxic, ex vivo-expanded autologous CD38^−/low^ NK cells are being investigated to alleviate NK cell fratricide during anti-CD38 antibody therapy [90, 91]. Moreover, KO of CD38 improves metabolism (glycolysis, oxidative phosphorylation, and oxidative stress resistance), function, and persistence of PB NK cells [91]. Higher frequency of activated/exhausted TIM3^+^ and HLA-DR^+^ NK cells has been reported to be associated with daratumumab resistance and is predictive of inferior PFS and OS of daratumumab treatment [106]. Addition of healthy donor-derived purified NK cells can overcome both NK cell-mediated primary and acquired anti-CD38 antibody resistance and support the clinical evaluation of anti-CD38 antibodies combined with adoptive transfer of NK cells [106, 120]. Elotuzumab, another mAb, targets SLAMF7 on myeloma cells and induces ADCC by NK cells [109]. Elotuzumab in combination with ex vivo-expanded NK cells displays enhanced ADCC activity of NK cells against MM, and the combination is under clinical investigation in high-risk MM [121].

NK cell adoptive therapy employs autologous or allogenic ex vivo expansion of NK cells using cord blood (CB)-derived NK cells, induced pluripotent stem cells (iPSCs), PB, NK92 cell line, and CB hematopoietic stem/progenitor cells (HSPCs). For this high-risk symptomatic MM population, CBNK cells (autoSCT background) are a promising adjunct immunotherapy [122], and reinfusion of expanded NK cells could provide therapeutic benefits in MM [7, 14]; CAR-NK cell therapy is another type of adoptive NK cell therapy [7, 14, 115]. Although CAR therapy focuses on T cells because of clinical efficacy, CAR-T therapy has several shortcomings (cytokine-release syndrome [CRS], neurological toxicities, and inefficiencies associated with T cell isolation, modification, and expansion), which support the development of CAR-NK therapeutics [14]. CAR-NK therapy is being considered an attractive platform for off-the-shelf cell therapy, including CAR-NK therapy focused on donor-derived or iPSC NK cells; it also provides a solution for overcoming manufacturing challenges, i.e., autologous cell material and safety profile. Moreover, CAR-NK therapy holds several advantages over allogeneic CAR-T therapy, including no risk of graft-versus-host disease (GvHD), no requirement for HLA matching, and minimal CRS risk. A Phase I/II trial involving umbilical cord-derived CAR-NK cells for R/R CD19^+^- lymphoid tumors resulted in responses for majority of patients without any major toxic effects [123].

Engineered “feeder” cells express ligands that activate NK cells and are used with cytokines, such as IL-2 and IL-15, for the maintenance and expansion of healthy, cytotoxic NK cells [124]. IL-15 is involved in NK cell differentiation, survival, and proliferation. IL-15 priming increases granzyme B protein levels in CD56^bright^ NK cells [76]. Cytokine-induced memory-like (CIML) NK cells, another option for allogeneic cell therapy, have unique advantages over other NK cell products. These cells are generated ex vivo through brief priming with IL-12, IL-15, and IL-18, which yields NK cells with enhanced and long-lasting responsiveness to cytokines and activating receptor stimulation [14, 124].

### Other NK cell activators and approaches to enhance the potency of different NK therapeutics

Recently, IL-15 agonists, such as ALT-803 and NKTR-255, have been investigated for ADCC enhancement. ALT-803, an IL-15 super agonist fusion protein, enhances NK cell-mediated ADCC and stimulates NK cells and CD8^+^ T cells [125]. NKTR-255, a polymer-conjugated IL-15 receptor agonist, augments daratumumab-induced ADCC [126]. Moreover, an anti-CD137 agonist mAb, urelumab, demonstrated synergistic activity with daratumumab in a myeloma mouse model reconstituted with human NK cells [127]. Venetoclax, a B-cell lymphoma 2 (Bcl-2) inhibitor under development, showed NK cell-mediated ADCC enhancement with CD38 antibody in Bcl-2 overexpressing (11:14) cell lines [128]. Venetoclax selectively induces NKG2DLs expression and shows synergistic cell cytolysis activity with NK cells [129].​ Thus, Bcl-2 inhibitors could be combined with NK-cell-based therapies.

ROCK (Rho-associate protein kinase) inhibitors represent another interesting class of molecules. A small molecule screen identified ROCK as a regulator of NK cell cytotoxicity against cancer cell lines [130]. Interestingly, belumosudil, a ROCK2 inhibitor approved for GVHD also showed antimyeloma activity alone and in combination with isatuximab [131]. A recent report suggested that IKAROS and AIOLOS play crucial roles in NK cell development. Targeting the AP-1-IRF transcriptional complex without affecting IKAROS and AIOLOS may offer differentiated pharmacology over the current IMiDs [112]. Belumosudil acts by degrading interferon regulatory factor 4 (IRF4); it would be interesting to see if the antimyeloma effect of belumosudil also translates into augmentation of NK cell activity and NK cell-based therapies in the future.

## Conclusions

NK cells are critical regulators of immune surveillance against malignant cells. NK cells display both cytotoxic and cytokine-producing properties as well as shape a multicellular immune response against tumor cells by involving DCs and T cells. NK cells and NK cell-based therapies are emerging as a safer and effective option in the MM therapeutic landscape. Thus, understanding NK cell biology and NK cell–MM interaction is crucial for supporting this new development.

To fully exploit the potential of NK cell-based immunotherapy, we need to understand the immune escape mechanisms of MM cells and the effects of the existing antimyeloma agents on NK cells. Development of combination strategies will require elucidation of the NK cell-augmenting properties of agents such as IMiDs and cereblon E3 ligase modulatory drugs (CelMODs) and optimal sequencing of antimyeloma agents such as PIs. Strategies to combine ADCC-dependent antimyeloma antibodies with allogeneic NK cells and ADCC-enhancing small molecules will play an important role in this context.

Current MM drug therapies exert their therapeutic effects in part via NK cells but could also contribute to exhaustion/dysfunction. IMiDs and cytokines (e.g., IL-2, IL-15) may enhance the function of NK cells. Optimal response post ASCT and daratumumab therapy requires sufficient BM infiltration by functional NK cells. For optimal efficacy of NKCEs, they should be investigated in combination with allogeneic NK cells and/or cytokines.

Adoptive NK cell transfer is another emerging area where optimal lympho-depleting regimen, disease state, disease burden, time of treatment, and persistence and expansion of infused NK cells play an important role. Thus, optimization of these factors will unravel new therapeutic options. NKCEs also present a promising new treatment strategy. Important preclinical activity of NKCEs has been demonstrated against BCMA-expressing MM cells, and early phase data are awaited [117]. Combinations of NKCEs with allogeneic NK cells and cytokines have shown tremendous potential [132]. Genetic modification of NK cells, such as CAR-NKs and CISH-KO or CD38-modulated NK cells, provides additional functions to NK cells. Thus, NK cell-based therapies could be an effective and safer therapeutic option for MM, with the potential of being combined with several other modalities and for improving outcomes of patients with MM.

## Data Availability

No datasets were generated or analysed during the current study.

## Abbreviations

A2A: Adenosine-purinergic adenosine
ADCC: Antibody-dependent cell-mediated cytotoxicity
BCMA: B cell maturation antigen
BiKEs: Bispecific killer cell engagers
BM: Bone marrow
CAR: Chimeric antigen receptor
CB: Cord blood
c-Cbl: Casitas B-lineage lymphoma
CD: Cluster of differentiation
CRBN: Cereblon
CRS: Cytokine-release syndrome
EAT-2: EWS-activated transcript 2
ER: Endoplasmic reticulum
ERK: Extracellular regulated MAP kinase
ERp5: Endoplasmic reticulum protein 5
FasL: Fas ligand
GITR: Glucocorticoid-induced TNFR-related protein
GvHD: Graft-versus-host disease
HLA-I: Human leukocyte antigen-1
HSCT: Hematopoietic stem cell transplantation
ICD: Immunogenic cell death
ICOS: Inducible T cell costimulatory
IFN-γ: Interferon-γ
IMiDs: Immunomodulatory drugs
ILCs: Innate lymphoid cells
IL: Interleukin
IRF4: Interferon regulatory factor 4
KIRs: Killer immunoglobulin like receptors
iPSCs: Induced pluripotent stem cells
mAbs: Monoclonal antibodies
MAPK: Mitogen-activated protein kinase
MGUS: Monoclonal gammopathy of undetermined significance
MM: Multiple myeloma
MRD: Minimal residual disease
MSCs: Mesenchymal stromal cells
NDMM: Newly diagnosed multiple myeloma
NKCEs: NK cell engagers
NK: Natural killer
PB: Peripheral blood
PD-1: Programmed cell death protein 1
PGE2: Prostaglandin E2
PIs: Proteasome inhibitors
PLCγ: Phospholipase C gamma 1
p-MHC I: Peptide-major histocompatibility complex-I
PRF1: Perforin
ROCK: Rho-associate protein kinase
RRMM: Relapsed/refractory multiple myeloma
SLAMF: Signaling lymphocytic activation molecular family 7
SMAD3: SMAD family member 3
sMICA: Soluble MICA
SMM: Smoldering multiple myeloma
TRiKEs: Trispecific killer engagers
TIGIT: T-cell Ig and ITIM domain
TIM-3: T cell immunoglobulin and mucin domain-3
TNF-α: Tumor necrosis factor-α
TME: Tumor microenvironment
